# Extrathyroidal Expression of the Thyrotropin Receptor in Epithelial Cancer Cells

**DOI:** 10.3390/ijms27198487

**Published:** 2026-09-23

**Authors:** Nasreen Nakad, Rinat Bar-Shalom, Naiel Azzam, Fuad Fares

**Affiliations:** Department of Human Biology, Faculty of Natural Sciences, University of Haifa, Haifa 3498838, Israel

**Keywords:** thyroid-stimulating hormone receptor, thyrotropin receptor, epithelial cancer, thyrostimulin, missense mutations, extrathyroidal expression, tumor progression

## Abstract

Glycoprotein hormone receptors are G-protein-coupled receptors essential for endocrine signaling. Thyroid-stimulating hormone receptor (TSHR), originally expressed in the thyroid gland, has been increasingly detected in various normal and malignant tissues, suggesting a wide range of functions. We hypothesized that extrathyroidal TSHR contributes to tumor development and activation via local stimulators such as thyrostimulin and its subunits. In the present study, TSHR expression was characterized in breast (T47D, MCF7), pancreatic (PL45, BXPC3), and colon (HCT116) cancer cell lines using quantitative real-time PCR, immunohistochemistry, and Western blotting. The sequencing of the TSHR cDNA revealed several novel mutations with potential functional relevance. Notably, missense mutations, Glu362Asp (PL45, HCT116) and Ala380Pro (T47D) were identified in the extracellular hinge region, which is a key domain for ligand binding and receptor activation. Functional interpretation suggests that Glu362Asp may lower the steric barrier, thereby enhancing the receptor sensitivity to non-canonical ligands such as thyrostimulin. In addition, Ile419Leu identified in T47D cells and located within the transmembrane Helix 1 may alter receptor conformation and signaling capacity. Consistent with these findings, variable proliferative responses to bovine thyroid-stimulating hormone (bTSH) were observed across the examined cell lines. Collectively, these results functionally identify relevant TSHR variants in cancer cells and support a model in which extrathyroidal TSHR signaling contributes to tumor progression. Targeting these receptors may represent a novel therapeutic approach.

## 1. Introduction

Thyrotropin receptor, also known as the thyroid-stimulating hormone receptor (TSHR), is a part of the glycoprotein hormone receptor family and is defined as a class of G-protein-coupled receptor (GPCR) [1]. Structurally, TSHR is a transmembrane receptor composed of 764 amino acids. Its large, highly glycosylated extracellular domain (ECD) contains a leucine-rich domain (LRD), which serves as the binding site for TSH and autoantibodies, and [2,3] a hinge region that plays an important role in ligand binding affinity and signal transduction and has also been implicated in ectodomain mediated inverse agonist activity [2]. The hinge region, acting as a flexible linker, connects the LRD to the seven-helix transmembrane domain (TMD), which, together with the intracellular loops and C-terminus, mediates downstream signaling [3,4].

TSHR was initially thought to be functionally restricted to the thyroid gland. However, accumulating evidence has demonstrated its expression in a variety of normal and malignant human tissues and cell models beyond the thyroid gland. These include bone marrow [5], adipose tissue [6], thymus [7], brain, liver [8] and hepatocellular carcinoma [9], ovarian cancers [10,11,12] and breast cancer [13]. These findings suggest that TSHR has broader physiological and pathological roles beyond thyroid hormone regulation. Pathogenic involvement of TSHR has been extensively investigated in autoimmune thyroid diseases. In autoimmune thyroid diseases, TSHR serves as a primary target, as it is targeted by both antibodies and antigen-specific T cells [14]. In autoimmune hyperthyroidism, such as Graves’ disease, the predominant cause of hyperthyroidism, stimulating autoantibodies act as an agonist of TSH, leading to constitutive activation of TSHR signaling [1]. In contrast, in autoimmune hypothyroidism, such as Hashimoto’s thyroiditis, autoantibodies function as antagonists, inhibiting receptor activity [14].

The effects of TSHR mutations on the receptor function and clinical presentation have been studied over the years, where both gain- and loss-of-function mutations were found to have distinct effects. Loss-of-function (inactivating) mutations, most frequently identified in exon 10, underlie a spectrum of TSH resistance phenotypes ranging from severe non-autoimmune hypothyroidism to mild hyperthyroxinemia with preserved thyroid function [15,16]. The extent of resistance to TSH is correlated with the level of receptor dysfunction, caused by both the nature of the mutation and the number of mutant alleles present [17,18].

Activating mutations in TSHR on the other hand result in a constitutively activated receptor, even in the absence of a ligand, being the cause of a wide range of thyroid gland syndromes. These include somatic mutations that are a primary cause of benign toxic thyroid adenomas, and may also contribute to the development of certain multinodular goiter cases [19]. Similar activating mutations on the TSHR have been identified as the underlying cause of several conditions, including autonomous adenomas, familial nonautoimmune hyperthyroidism, and sporadic congenital nonautoimmune hyperthyroidism, all of which exert comparable effects on the thyroid gene expression and physiology [20]. Collectively, these disorders fall within the spectrum of genetic hyperthyroidism, with phenotypic differences determined by the strength of receptor activation, the timing of mutation during development, and the range of affected cells [20]. Furthermore, the expression of extrathyroidal TSHR in normal and cancer cells has been investigated in the past few years. For instance, its expression has been studied in liver cells because of a possible link between TSH levels and hepatocyte’s function. This was based on observation of a direct correlation between circulating TSH levels and the production of hepatic C-reactive protein (CRP) in subclinical hypothyroidism patients [21,22]. Subsequently, Zhang et al. (2009) demonstrated that the normal human hepatocyte cell line, L-02, expresses a TSHR-associated activity at mRNA and protein levels when stimulated by bovine TSH leading to elevation in cAMP levels [8].

As for hepatocellular carcinoma (HCC), elevated TSHR expression has been associated with clinical outcomes, where high expression levels were correlated with poor patient survival [9,23]. The sensitivity of patients to the TSHR signaling changed the response to the chemotherapeutic agent, cisplatin, in vitro [9]. Analysis of the TSHR coding sequence revealed multiple mutations located in exon 10. However, their impact on cancer progression in HCC remains unclear [23].

Extrathyroidal TSHR expression was also well studied in ovarian cancer, where TSHR expression has been demonstrated in human ovarian tissues by both immunohistochemical staining and RT-PCR [11]. Its activation by TSH was confirmed through the analysis of downstream signaling pathways and metabolic outputs [11]. In addition, the receptor expression in ovarian cancer tissue increased as a result of cAMP signal induced by gonadotropins and decreased in response to estradiol production in rat ovaries [10,12,24]. Nevertheless, former studies indicated that the primary activator of TSHR is the local ligand thyrostimulin rather than TSH [10,12].

Over the past two decades, locally produced molecules, including thyrostimulin and TSHꞵ variant (TSHβ_V_), have been identified as activators of TSHR signaling in peripheral tissues, suggesting their roles in local tissue function and development. Consistent with this, TSHR activation has been implicated in the regulation of tissue development and repair. For example, mesenchymal stem cells derived from human bone marrow have been shown to express TSHR, and the exposure to recombinant TSH enhanced their differentiation to the chondrogenic lineage [5]. Furthermore, TSHR activation by thyrostimulin may play an important role in transient developmental processes, including the regulation of differentiation and self-renewal and survival of progenitor and stem cell populations [25,26,27].

Despite this expanding landscape, the extrathyroidal expression, mutational status and functional role of TSHR in epithelial cancers remain poorly characterized. Here, we investigated TSHR expression in pancreatic (PL45, BXPC3), colon (HCT116) and breast (T47D, MCF7) cancer cell lines and compared them with CHO cells expressing or lacking TSHR and with normal human dermal fibroblasts (NHDF). We quantified TSHR mRNA and protein levels, assessed cellular localization, evaluated the effect of bTSH on cell viability, and sequenced TSHR cDNA to identify coding variants. In addition, we used AlphaFold-based structural modeling and visualization to explore potential structural consequences of selected TSHR variants.

## 2. Results

### 2.1. TSH Receptor mRNA in Cancer Cells

To determine TSHR expression across epithelial cancer cell lines, total RNA was extracted from PL45, BXPC3, HCT116, T47D and MCF7 cells, as well as from non-transfected CHO cells, TSHR-transfected CHO cells and NHDF cells. CHO cells transfected with the human TSHR gene served as a positive control, non-transfected CHO cells served as a negative control, and NHDF cells were included as a normal cell line. cDNA was amplified using specific primers for TSHR (Table 1). The overall differences among cell lines were initially evaluated using the Kruskal–Wallis test, followed by comparison between each cell line and the CHO cell controls using Mann–Whitney U test with Bonferroni correction for multiple comparisons. A significant elevation in TSHR expression was observed in PL45, BXPC3, HCT116, T47D, and MCF7 cells compared with the negative control non-transfected CHO cells (Mann–Whitney U = 0.00, Z = −3.08, *p* = 0.002) and identical Mann–Whitney values were observed for all tested cell lines. Similarly, significant increase in the TSHR mRNA levels was observed in CHO cells overexpressing the TSHR compared with the negative control CHO cells (Mann–Whitney U = 0.00, Z = −2.88, *p* = 0.004) as shown in Figure 1. The expression levels of TSHR increased by 81.4 ± 20.3, 20.4 ± 4.9, 66.7 ± 16.5, 164.8 ± 54.3, 203.4 ± 39.6, 9569.6 ± 1435.6 in PL45-, BXPC3-, HCT116-, T47D-, MCF7- and TSHR-transfected CHO cells respectively. Data presented are the average of at least three independent experiments, each conducted in triplicates (mean ± SE) and expressed as percentage of the respective control (negative CHO cells).

Immunofluorescence staining (IF) was used to assess TSHR subcellular localization. TSHR immunoreactivity (red signal) was predominantly detected along the plasma membrane in PL45, BXPC3, HCT116, T47D and MCF7 cells, whereas NHDF cells showed minimal staining (Figure 2). These findings are consistent with the presence of cell-surface TSHR in the cancer cell lines.

### 2.2. The Effect of TSH on Metabolic Activity in Human Cancer Cells

NDHF (non-cancerous fibroblast), the cancer cells found to express TSHR; PL45, BXPC3, HCT116, T47D and MCF7, were grown in the absence or presence of 100 ng/mL bovine (bTSH) with or without addition of TSHR antagonist 3 µg/mL (ML224) for 48 h and cell viability was detected. The use of NHDF cells in this study was intended primarily as a non-malignant human cell control and not as a tissue-matched normal counterpart for the different cancer cell lines. Post hoc Bonferroni analysis revealed that the bTSH significantly increased cell viability compared with the untreated control (*p* < 0.001), whereas TSHR antagonist, ML224, significantly (*p* < 0.031) reduced/inhibited the bTSH stimulated viability (Figure 3).

Two-way ANOVA revealed a significant overall effect of bTSH and bTSH in combination with ML224 treatments on cell viability (F(2, 41) = 81.16, *p* < 0.001). Furthermore, a significant effect of the treatments (bTSH and bTSH-ML224) with the cell type interaction in each cell type was examined. Subsequently, one-way ANOVA analysis performed for each cell line. In NHDF cells no significant effect was detected of the treatments on cell viability (2, 6) = 1.669, *p* = 0.265. In TSHR-transfected CHO cells, one-way ANOVA revealed a significant effect of the treatments on cell viability F(2, 6)= 31.029, *p* < 0.001. Bonferroni post hoc analysis showed that bTSH significantly increased viability compared with both control (*p* = 0.002) and ML224 (*p* = 0.001), whereas ML224 did not differ significantly from control (*p* = 1.00). Also, in PL45, BXPC3, HCT116, T47D, and MCF7 cells, one ANOVA revealed the significant effects of treatment on cell viability: F(2, 6) = 7.86, *p* = 0.021; F(2, 6) = 540.08, *p* = 0.000; F(2, 6) = 15.90, *p* = 0.004; F(2, 6) = 36.954, *p* = 0.001; F(2, 6) = 11.216, *p* = 0.014.

Whereas a significant increase in cell viability following bTSH treatment was detected, in PL45 (12.7 ± 3.32%)-, BXPC3 (13.2 ± 0.44%)-, HCT116 (11.84 ± 3.1%)-, T47D (14.7 ± 1.75%)-, MCF7 (16.9 ± 4.16%)- and TSHR-transfected CHO cells (26.73 ± 4.21). Moreover, treatment with ML224 significantly reduced cell viability induced by bTSH in BXPC3, HCT116, T47D and MCF7 cells expressing TSHR (*p* < 0.031), whereas no significant effect was observed in NHDF and PL45 cells.

### 2.3. TSHR Mutation in Cancer Cell Lines

TSHR cDNAs sequences divided into fragments were amplified from cDNA derived from PL45, T47D, and HCT116 cell lines using specific primers (Table 2), followed by reverse-transcription of total RNA using oligo(dT) Primers.

Sequence analysis of the amplified cDNA fragments using specific primers (Supplementary Figure S1) revealed several mutations, identified by comparison with the WT TSHR sequence using the NCBI BLAST database. This analysis allowed us to identify and characterize the nucleotide differences relative to the WT TSHR sequence. In PL45 cells, a missense mutation located in exon 10 was detected at c.1086 G > T (Glu 362 Asp). In addition, one silent mutation was identified at position c. 1257 T > A (Ile 419 Ile). In T47D cells, three different mutations were identified, all in exon 10 at positions c. 1138 G > C (Ala 380 Pro), c. 1255 A > C & c. 1257 T > A (Ile 419 Leu), and c. 2181 C > G (Asp 727 Glu). As for HCT116 cells, two missense and silent mutations were detected on exon 10, including c.1086 G > T (Glu 362 Asp), and c.1650 C > T(Phe550Phe), respectively (Table 1).

### 2.4. TSHR Structural Prediction in Cancer Cells’

The AlphaFold predictions of WT-TSHR and mutated TSHR found in cancer cells structures were performed using AlphaFold 3 Server [28]. The structures shown in Figure 4 are colored according to the predicted local Distance Difference Test (pLDDT) confidence scores. Regions colored in dark blue represent very high prediction confidence (pLDDT > 90), whereas light blue indicates high confidence (70–90). Yellow regions correspond to low confidence (50–70), and orange to red regions represent very low confidence (pLDDT > 50). Overall, the transmembrane helices and core of the receptor exhibited high prediction confidence, while the extracellular loops, intracellular regions, and terminal segments displayed lower confidence, suggesting increased structural flexibility in these regions.

### 2.5. Structural Visualization and Differences Produced by Mutagenesis

#### 2.5.1. Ile 419 Leu, Ala380Pro and Asp727Glu Mutations in T47D Cells

##### Ile 419 Leu Mutation

Comparative structure analysis of the AlphaFold predicted WT TSHR and mutant Ile 419 Leu variant identified in T47D cells (Figure 5) revealed a local conformational difference within the first transmembrane alpha-helix backbone. Although Isoleucine Leucine are structurally similar hydrophobic amino acids, the substitution of Ile419 with Leu419 may be associated with a change in side-chain orientation and a minor displacement of the local polypeptide backbone. Structural superimposition of the wild-type and mutant models yielded a backbone RMSD of 0.48 Å, indicating that the overall receptor structure remained highly conserved despite local conformational changes. These observations may suggest that I419L substitution may subtly alter the local packing of the transmembrane helix (Figure 5); however, the functional consequences of this variant require further experimental validation.

#### 2.5.2. Ala380Pro Mutation

In addition to the I419L substitution, T47D cells harbored the Ala380Pro (A380P) variant. Comparative structure analysis of the AlphaFold prediction AT and mutant TSHR models demonstrated a conserved structure core, with a backbone RMSD of 0.482 Å across 603 aligned Cα atoms. In contrast, global alignment of all 764 Cα atoms yielded an RMSD of 14.932 Å, and the corresponding Cα atoms at residue 380 were separated by 46.856 Å (Figure 5). However, AlphaFold assigned low confidence to residue 380 in both the WT (pLDDT ≈ 24.6) and mutant (pLDDT ≈ 30.8) models. Therefore, although the predicted structures differ substantially at this position, these local conformational differences require extra experimental validation.

#### 2.5.3. Asp727Glu Mutation

Comparative structural analysis of the AlphaFold prediction of the WT-TSHR and the mutant Asp727Glu variant identified in T47D cells revealed only minor local conformational differences within the intracellular C-terminal region of the receptor. The substitution of the aspartic acid with glutamine acid represents a conservative amino acid change as a result of the similar physicochemical properties and negative charge. The position of the structure demonstrated a minor ordination of the side chain and the surrounding backbone with overall highly conserved structure. Results are consistent with the conservation of this mutation as it is reported in the literature as this mutation is properly inducing minor structure rearrangements. However, as is reported, this polymorphism is associated with an enhanced cAMP response when activated by TSH in certain conditions [29], indicating that it has a biological effect on changing the receptor signaling even though the structural effect was minor therefore further experimental investigations are needed to clarify the effect.

#### 2.5.4. Glu362Asp Mutation in PL45 and HCT116 Cells

Comparative structural analysis of the AlphaFold predicted WT TSHR and the Glu362Asp variant identified in PL45 and HCT116 cells revealed subtle local conformational differences within the hinge region of the receptor (Figure 6). The substitution of glutamic acid with aspartic acid at position 362 results in the reduction in the side-chain methylene length while preserving its negative and overall physicochemical properties. Structural superimposition demonstrated a local backbone displacement of approximately 0.506 Å, indicating a modest conformational adjustment associated with the amino acid substitution. Surface topology analysis revealed localized differences in the molecular surface surrounding residue 372, and the volumetric analysis of the predicted structure showed a Minor reduction in total molecular volume of the mutants compared with the WT (TSHR WT: 1.001 × 10^5^ Å, mutant: 9.997 × 10^4^ Å). These findings suggest that the E362D substitution may alter the local structural environment while preserving the overall architecture of the receptor.

Notably, AlphaFold assigned low confidence to residue 362 in both the WT and mutant models (average pLDDT 30), indicating that the local conformation of the region is predicted with limited certainty. Consequently, the observed structure differences should be interpreted cautiously and require experimental validation to determine their functional significance.

## 3. Discussion

In this study, we provide evidence for extrathyroidal expression of TSHR-associated activity in pancreatic, colon and breast cancer cell lines, and we identify several TSHR coding variants that may influence receptor structure and signaling in a cell-type-specific manner. These observations expand earlier reports of TSHR in hepatocytes, HCC, ovarian tissue and breast cancer [8,9,10,11,12,13,23,24] and support a broader role for TSHR in epithelial tumor biology.

TSHR has long been implicated in thyroid tumorigenesis, where activating mutations and TSH-dependent signaling can promote cell viability and hormone production. Furthermore, spontaneously accruing mutations in the TSHR have the ability to constitutively activate signaling pathways, thereby inducing cell proliferation. Such mutations include V509A, C672Y, and M453T [23].

No studies to date have reported TSHR expression in human pancreatic cancer. However, TSHR expression has been demonstrated in rat pancreatic islets and in the rat pancreatic β cell line (INS-1), where activation of TSHR by its ligand (TSH) stimulated the expression of glucose transporter 2 (GLUT 2), which directly affects the glucose uptake and increases insulin secretion [30]. Furthermore, comparative analyses of hyperthyroid and hypothyroid patients with euthyroid group showed elevated insulin levels in thyroid disorders, although insulin resistance (IR) values also increased [31].

TSHR expression in breast cells has been previously reported in both normal and malignant tissues, with elevated levels observed in tumor tissues compared with normal breast tissues. However, the biological significance of this elevation remains to be elucidated.

From a genetic perspective, both the receptor sequence and its functional characterization in epithelial cancer remain insufficiently understood [13,32]. In the present study, we identified multiple mutations within the TSHR coding sequence, all located in exon 10, the largest exon which encodes for the seven-transmembrane domain and the intracellular domains of the TSHR [33]. These domains are crucial for the receptor activation and signal transduction, leading to the activation of different signaling pathways, resulting in cell proliferation, synthesis and secretion of thyroid hormones [33]. Therefore, sequence alterations in this region may significantly impact receptor conformation and downstream signaling. Among the variants identified in the present study, the Asp727Glu polymorphism detected in the T47D cell line is a trans-version point mutation located within the intracellular portion of TSHR. This variant has been previously reported by Van Deure W.M et al. (2008) [34]. It was shown that Asp727Glu polymorphism is associated with normal FT4 levels despite reduced TSH levels, as well as increased bone mineral density (BMD) at the femoral neck [34]. This supports the notion that the Glu727 allele confers increased functional activity of TSHR [33,34]. Consistent with this, an in vitro study by Gabriel et al. (1999) [35] in COS-7 eukaryotic cells has demonstrated that the Glu-727 variant induces the production of higher levels of cAMP following TSH stimulation. However, the functional effect of polymorphism may appear to be context-dependent. Notably, Sykiotis et al. (2003) reported that Asp727Glu alone did not elevate cAMP levels, whereas its combination with Ala593Asn polymorphism results in constitutive activation of the cAMP signaling cascade [29,34,35]. In addition to this known mutation, we identified three novel missense mutations in TSHR that have not been previously reported. The Glu362Asp mutation was identified in PL45 and HCT116 cell lines and the Ala380Pro mutation in T47D cells, both located in the extracellular hinge region. In this region, spanning amino acid positions 289–409 play a crucial role in ligand binding, antibody interaction and signal transmission [4,36,37]. The third mutation, Ile419Leu, identified in T47D cells, is located within Transmembrane Helix 1 (TMH1). Notably, a nearby constitutively activating mutation, Val421Ile, that covers the allosteric binding pocket of the receptor and has been reported to modulate receptor conformation, promoting transitions between inactive and active states [4].

To further investigate the structural implications of these mutations, we performed in silico modeling using Google DeepMind AlphaFold Server and UCSF ChimeraX v1.8 in order to structurally visualize and predict the mutation effects found in T47D, PL45 and HCT116 cells. Further study of the Glu362Asp mutation identified in the PL45 /HCT116 hinge region, revealed that the mutant site exhibited a reduction of 330Å in the molecular volume when compared with the WT. These results could affect the enhanced As indicated by various thyrostimulin studies demonstrating its higher binding affinity for TSHR compared to TSH [12,38,39]. Two silent mutations were also identified. One was detected in PL45 cells at codon 419, and the other was detected in HCT116 cells at codon 550. In both cases, the nucleotide substitution did not alter the encoded amino acid sequence, resulting in the retention of isoleucine at position 419 and phenylalanine at position 550, respectively.

Our functional assays indicate that bTSH enhances cell viability in TSHR-positive cancer lines, yet we cannot exclude contributions from off-target effects or other signaling pathways. The lack of significant ML224 antagonism in PL45 cells, despite detectable TSHR expression, suggests that bTSH-induced viability in this line may depend less on TSHR or may involve compensatory mechanisms. Future work should distinguish between proliferation and survival, examine cell-cycle distribution and apoptosis, and explore downstream signaling cascades, including cAMP/PKA, PI3K/AKT and MAPK pathways.

This study has several limitations that should be considered when interpreting the findings. First, the expression of TSHR was assessed using qRT-PCR, Western blotting, and immunofluorescence; however, the specificity of the detected protein signal and the presence of TSHR at the plasma membrane were not independently confirmed by genetic loss-of-function or ligand-binding experiments. Therefore, the protein and localization data should be interpreted as evidence of TSHR immunoreactivity rather than definitive proof of receptor abundance or surface functionality. Second, the CCK-8 assay primarily reflects cellular metabolic activity and cannot, by itself, distinguish increased proliferation from enhanced survival or altered cellular metabolism. Accordingly, the effects of bTSH are described here as increases in CCK-8-measured cell viability or metabolic activity rather than as direct evidence of proliferation. Finally, the functional consequences of the identified sequence variants were inferred from sequence analysis and structural modeling and were not directly tested experimentally. These variants should therefore be regarded as candidate or potentially relevant variants requiring further validation.

In conclusion, the present study identifies TSHR transcripts and TSHR immunoreactivity in the examined pancreatic, colon, and breast cancer cell lines. Treatment with bTSH increased cell viability under the experimental conditions used, and this response was reduced by ML224 in several cell lines. and that these receptors harbor context-specific variants in exon 10. While our structural modeling provides mechanistic hypotheses, definitive conclusions regarding the impact of individual variants on tumor behavior require targeted functional studies in relevant cellular and in vivo models. Future work should integrate TSHR expression, ligand availability, mutational status and downstream signaling readouts to clarify how extrathyroidal TSHR contributes to carcinogenesis and whether it can be exploited therapeutically [40,41,42].

Further studies using genetic receptor manipulation, direct signaling assays, ligand-binding analyses, validated proliferation or survival measurements and in vivo studies using animal models will be required to determine the biological significance of extrathyroidal TSHR in epithelial cancer.

## 4. Materials and Methods

### 4.1. Cell Cultures

PL45, BXPC3, HCT116, T47D and MCF7 cells (ATCC, Rockville, MD, USA) and CHO (dhfr-) (Chinese hamster ovarian cells) were kindly supplied by PROLOR Biotechnology, Israel. PL45, HCT116, and MCF7 were maintained in DMEM medium, while BXPC3 and T47D in RPMI medium. All mediums supplemented with 1% PenStrep, 1% L-glutamine, 1% sodium pyruvate according to the manufacturer’s instructions (Biological industries, Beit HaEmek, Israel) and 10% fetal bovine serum (FBS), (Sigma-Aldrich Israel Ltd., Rehovot, Israel). Furthermore, non-transfected CHO (dhfr-) and transfected CHO cells were maintained in Iscove’s media supplemented with 1% PenStrep according to the manufacturer’s instructions (Biological industries, Beit HaEmek, Israel) and 10% fetal bovine serum (FBS), (Sigma-Aldrich Rehovot Ltd., Rehovot, Israel), with the addition of 1% of HT Supernatant only to the non-transfected CHO (dhfr-). Cells were maintained in a humidified incubator at 37 °C in an atmosphere containing 5% CO_2_ and were replenished with fresh culture media twice a week. The cell lines were constantly tested for mycoplasma contamination using the EZ-PCR Mycoplasma Test Kit supplied by Biological Industries (Beit Haemek, Israel).

### 4.2. RNA Extraction and cDNA Synthesis

Total RNA was extracted and purified from PL45, BXPC3, HCT116, T47D and MCF7 cells, using RNeasy Mini Kit (QIAGEN, Petach Tikva, Israel) according to the kit protocol.

Total RNA was reverse-transcribed using the High-Capacity cDNA RT kit, (Applied Biosystems, Foster City, CA, USA) following the manufacturer’s instructions. Briefly, 2 µg of total RNA was reverse- transcribed using 2 μL 10 × Buffer, 2 μL Random Primers/oligo DT, 0.8 μL dNTP (10 mM), 1 μL MultiScribe Reverse Transcriptase, 1 μL RNase inhibitor and topped up with nuclease-free water to 20 μL. PCR reaction was performed at 25 °C for 10 min, 37 °C for 120 min and 85 °C for 5 min.

### 4.3. Real-Time PCR Analysis

A custom primer set designed for TSHR was used to quantify gene expression in cDNA derived from various cell lines. cDNA was produced using High-Capacity cDNA Reverse Transcriptase Kit (Applied Biosystems) according to the manufacturer’s instructions.

Quantitative PCR (qPCR) was performed using SYBR Green qPCR kit (Applied biosystems) following the manufacturer’s protocol. Amplification and data acquisition were carried out using the StepOne™ software v2.3 qPCR cycler.

Each experiment included three biological replicates, with each sample analyzed in triplicate (technical replicates). Relative gene expression levels across different cell lines and control CHO cells were determined using GAPDH (glyceraldehyde-3-phosphate dehydrogenase) as a housekeeping gene reference for normalization. Fold changes in gene expression were calculated using the 2^−∆∆Ct^ method, with NC serving as the reference control.

Melting curve analysis demonstrated a single dominant peak at approximately 83.4 °C in all experiments conducted using the specific TSHR primers, indicating specific amplification of the target amplicon with no evidence of significant nonspecific products. Furthermore, NTCs remained at baseline and did not exhibit a specific melting peak at the target melting temperature, confirming the absence of detectable contamination.

Non-transfected CHO cells were selected as the calibrator the human TSHR expression because they exhibited minimal TSHR expression and are widely used as control cells in TSHR-related studies. Furthermore, CHO cells represent the appropriate reference for comparison with TSHR transfects CHO cells included in this study. NHDF cells were additionally included as a normal human control and similarly demonstrated very low TSHR expression, comparable to CHO cells. Therefore, both of the following cell types provided a low-expression baseline for relative quantification of TSHR expression in the cancer cell lines.

### 4.4. Immunofluorescent Microscopy

Cells were seeded onto an 8-well chamber slide (Lab-Tek Chamber slide system, Nunc, Rochester, NY, USA) and cultured for 24 h. The cells were then washed three times with cold PBSX1, fixed with 4% formaldehyde for 20 min, and subsequently washed again.

The fixed cells were incubated and blocked with 10% normal goat serum for 30 min, thereafter treated at 4 °C for overnight with a dilution of 1:350 from the rabbit anti-TSHR polyclonal antibody (Bioss Antibodies Inc., Woburn, MA, USA). Three PBSX1 washes were conducted and followed by 1 h incubation with a 1:200 dilution of goat anti-rabbit Alexa Fluor 647- conjugated IgG and the Nuclei were stained with 4,6-diamidino-2-phenylindole (DAPI). And the immunofluorescence was viewed under a confocal microscope (Nikon A1R Confocal Laser Scanning Inverted Microscope, Tokyo, Japan). Negative control was detected on cells lacking the TSHR.

### 4.5. Sanger Sequencing

For TSHR gene sequencing, PCR amplification was performed using equal amounts of cDNA synthesized with oligo(dt) primers and specific primers for human TSHR (Table 1). The resulting PCR products were sequenced by Macrogen, Amsterdam, Netherlands. Obtained sequences were analyzed using Gene Runner and compared with the reference TSHR sequence using the NCBI BLAST database.

### 4.6. Structural Prediction and Mutational Analysis

Protein structure predictions for WT TSHR and the mutant receptor found in T47D,HCT116 cells were generated using the AlphaFold 3 Server engine throughout Google DeepMind AlphaFold Server using TSHR protein sequence [28].

### 4.7. Structural Visualization

Structural visualization and comparative analysis were performed using UCSF ChimeraX v1.8 [43]. The TSHR mutant found in T47D cells, and the WT receptor were superimposed to align the receptors and minimize the root-mean square deviation (RMSD) of the Cα backbone atoms.

### 4.8. Cell Viability Assay

The effect of bovine TSH (bTSH) (T8931; Sigma-Aldrich, St Louis, MO, USA) on cancer cell viability was performed using Cell Counting Kit-8 assay (CCK-8, APExBIO, Houston, Texas, USA), which measures cells viability. PL45, BXPC3, HCT116, T47D and MCF7 cancer cell lines were seeded at a density of 5000–8000 cells per well in 96- well plate. 24 h later cells were treated with 100 ng/mL bTSH with or without addition of TSHR antagonist 3 µg/mL (ML224). Following 48 h of treatment, 10 μL of the CCk-8 kit reagent was added to each well and incubated for 1–2 h. Absorbance was measured according to the manufacturer’s instructions, using a plate reader (BioTek, Winooski, VT, USA) at 450 nm wave. Experiments were performed independently three times. Data are presented as mean cell viability percentage normalized to the corresponding control.

### 4.9. Statistical Analysis

All experiments were independently repeated at least three times. Data are presented as mean value ± standard error (SE). Statistical differences among multiple groups were analyzed using one-way analysis of variance (ANOVA). Two-way ANOVA was performed to analyze the interaction between treatment and cell type. The value of *p* < 0.05 was considered statistically significant, and all statistical analyses were performed using SPSS software (IBM SPSS Statistics 26).

## Figures and Tables

**Figure 1:**
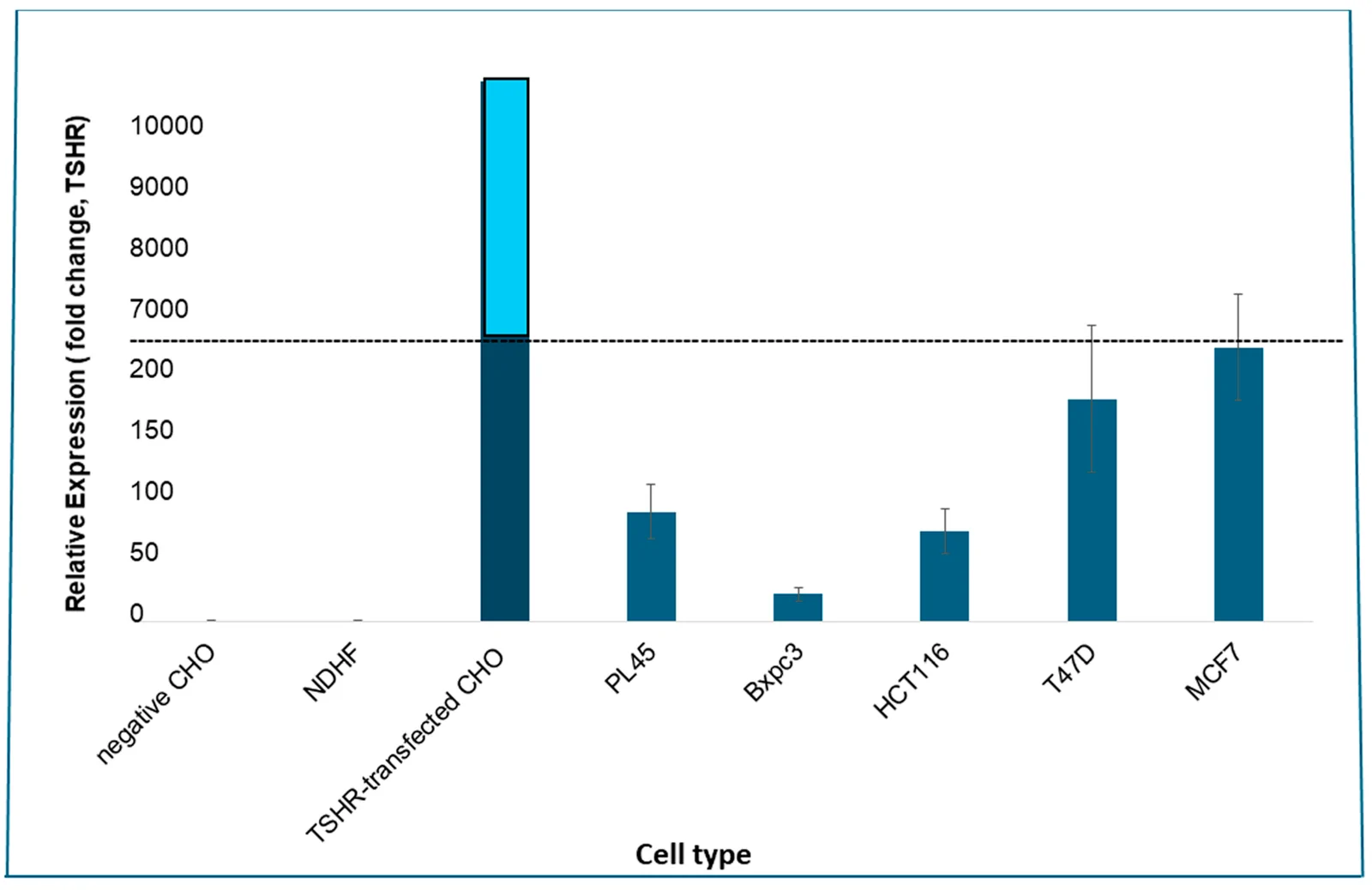
TSHR gene expression levels determined by Real time PCR. Total RNA was extracted from NHDF-, PL45-, BXPC3-, HCT116-, T47D-, MCF7-, and TSHR-transfected CHO cells, and negative CHO cells. negative CHO cells served as negative control. mRNA was reverse-transcribed into cDNA and used for real-time PCR using specific primers, as described under Section 4. A brake in the y-axis scale was introduced to accommodate the very high expression value of TSHR.

**Figure 2:**
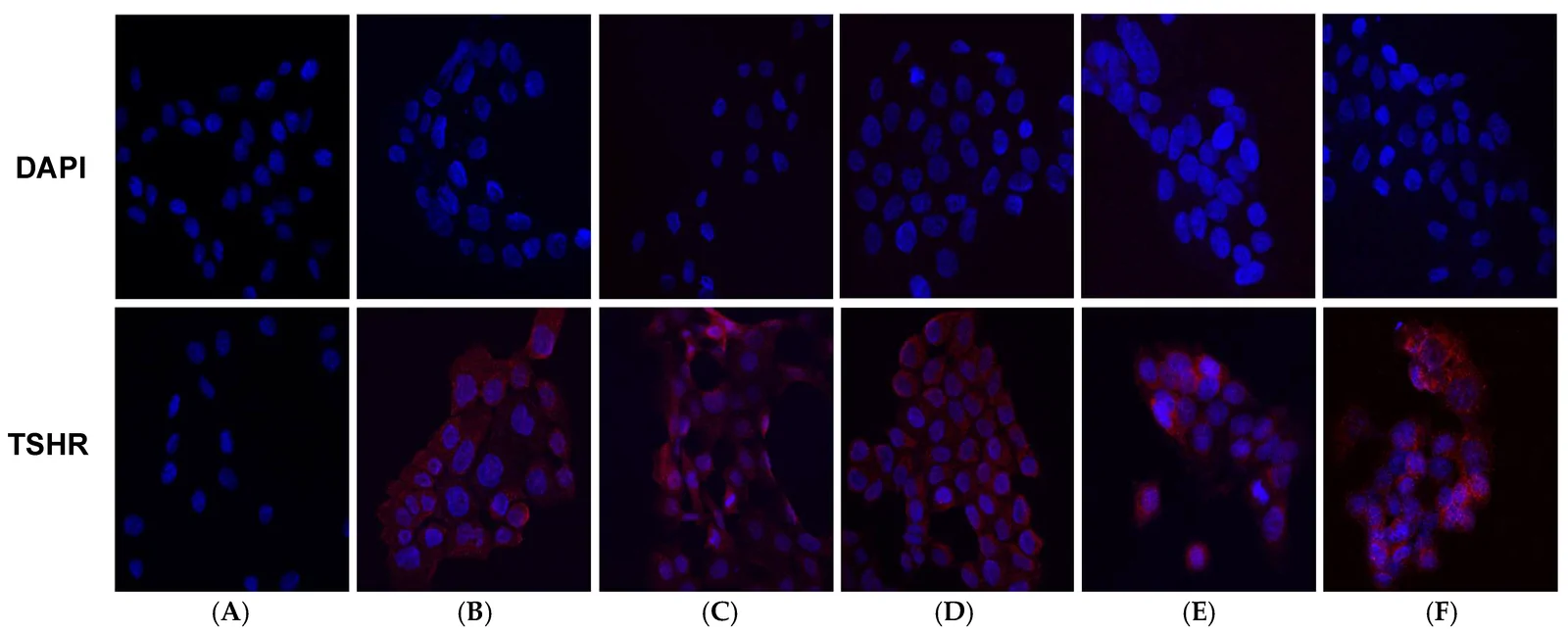
Immunofluorescence analysis of TSHR expression in NHDF (**A**), PL45 (**B**), BXPC3 (**C**), T47D (**D**), MCF7 (**E**) and HCT116 (**F**) cells. TSHR protein expression was detected using TSHR specific polyclonal antibody with Alexa Fluor™ 647 red. The fluorescence signal was predominantly localized to the plasma membrane, indicating membrane expression of TSHR. Cell nuclei were counterstained with DAPI (blue). Scale bars represent 20 μm.

**Figure 3:**
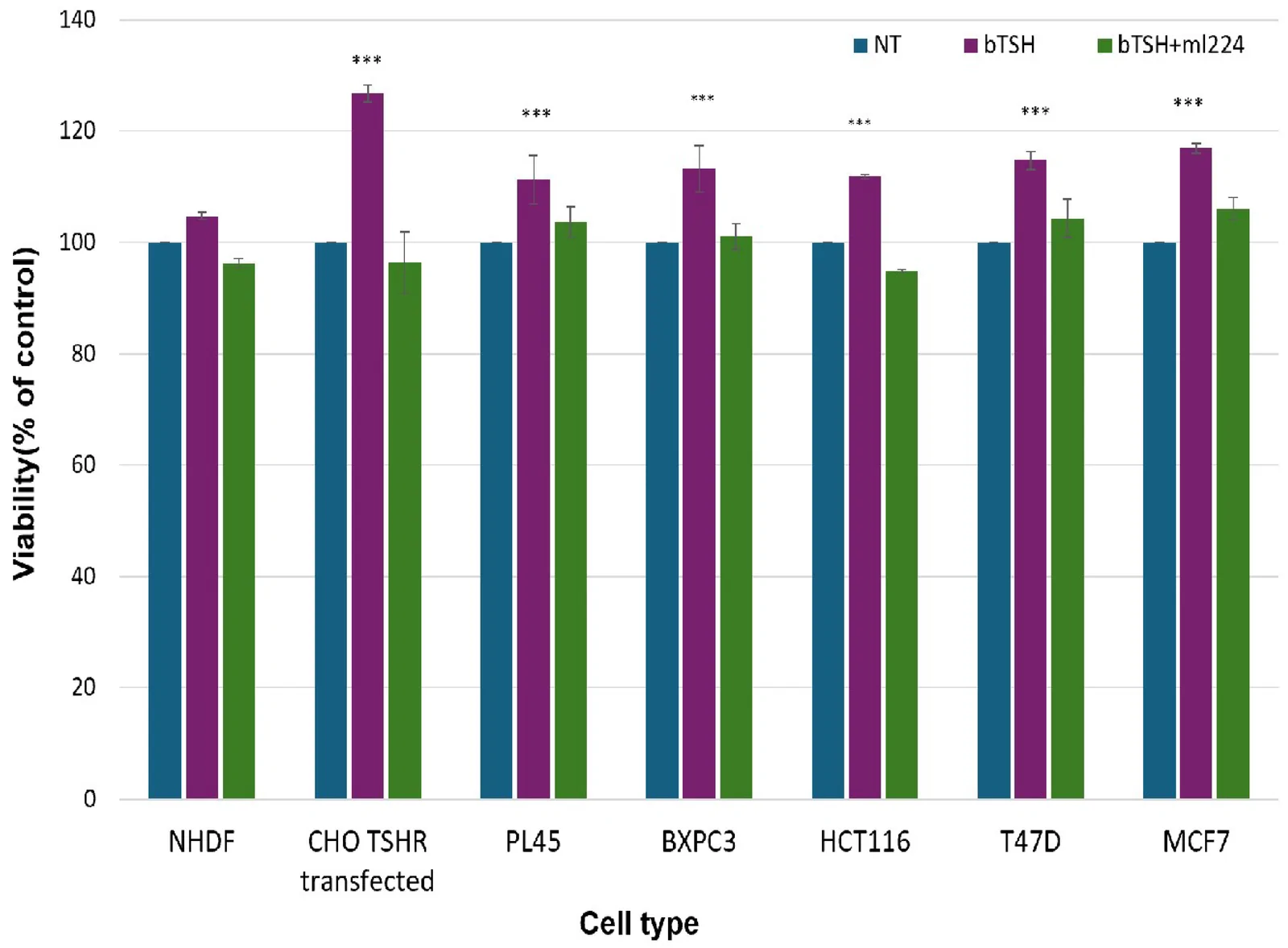
Effect of bTSH on metabolic activity. Cells were treated with 100 ng/mL of bTSH, in the presence or absence of TSHR antagonist, ML224, for 48 h. Metabolic activity was measured using CDK-8 assay as described under Section 4. Data are presented as the mean ± SE of three independent experiments each conducted in triplicates and are expressed as a percentage of the respective untreated control. Statistical significance was determined by one-way ANOVA with post hoc analysis for comparison treatments within each cell line, and two-way ANOVA to assess the interaction between treatment and cell type. Significance was determined as follows: three independent experiments, *** *p* < 0.001 versus untreated control.

**Figure 4:**
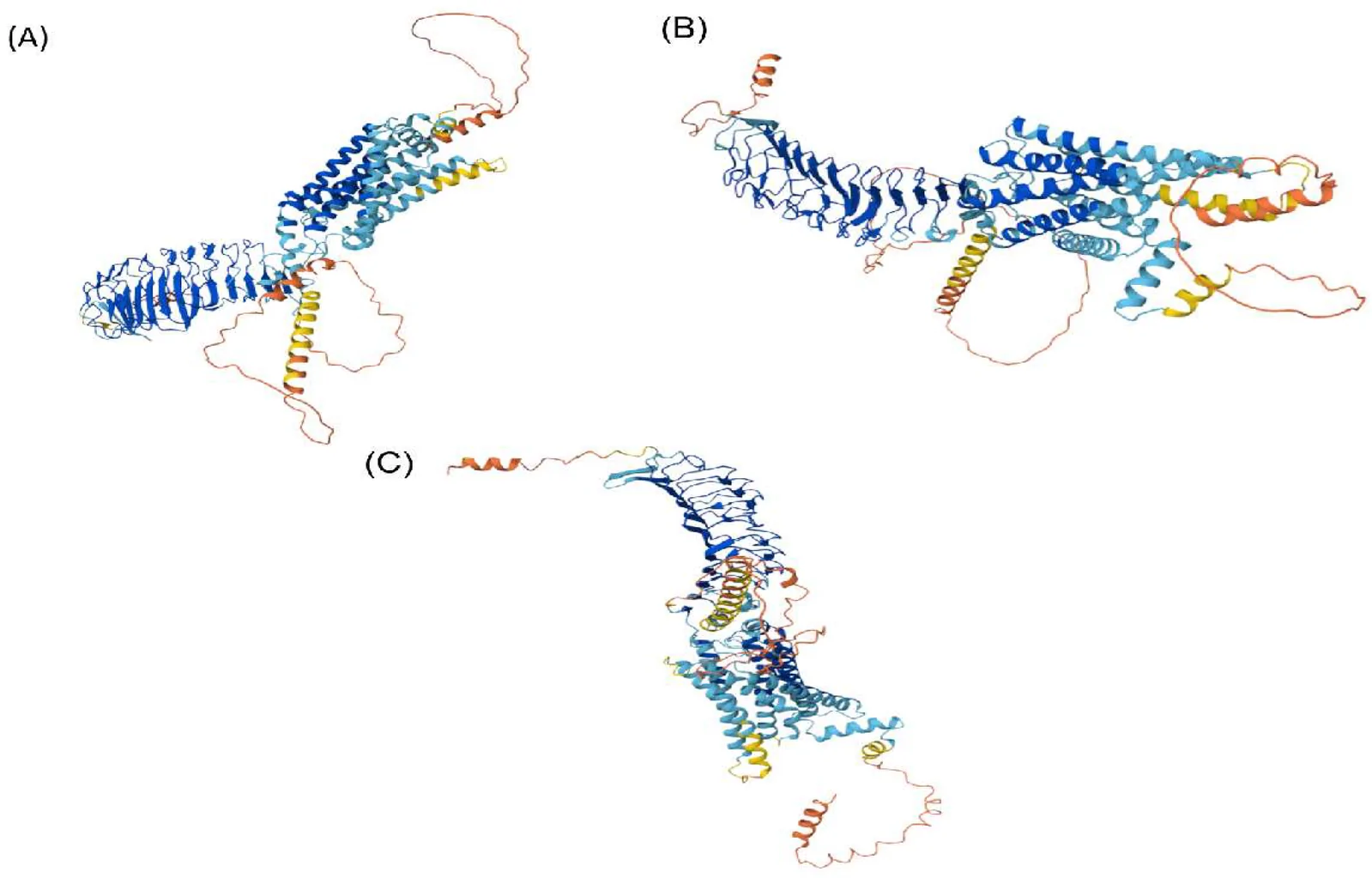
Protein structure predictions of WT-TSHR (**A**) Ile 419 Leu and Ala380Pro Mutation in T47D Cells (**B**) and TSHR Glu362Asp mutant In PL45 and HCT116 cells (**C**). Regions colored in dark blue represent very high prediction confidence (pLDDT > 90), whereas light blue indicates high confidence (70–90). Yellow regions correspond to low confidence (50–70), and orange to red regions represent very low confidence(pLDDT > 50).

**Figure 5:**
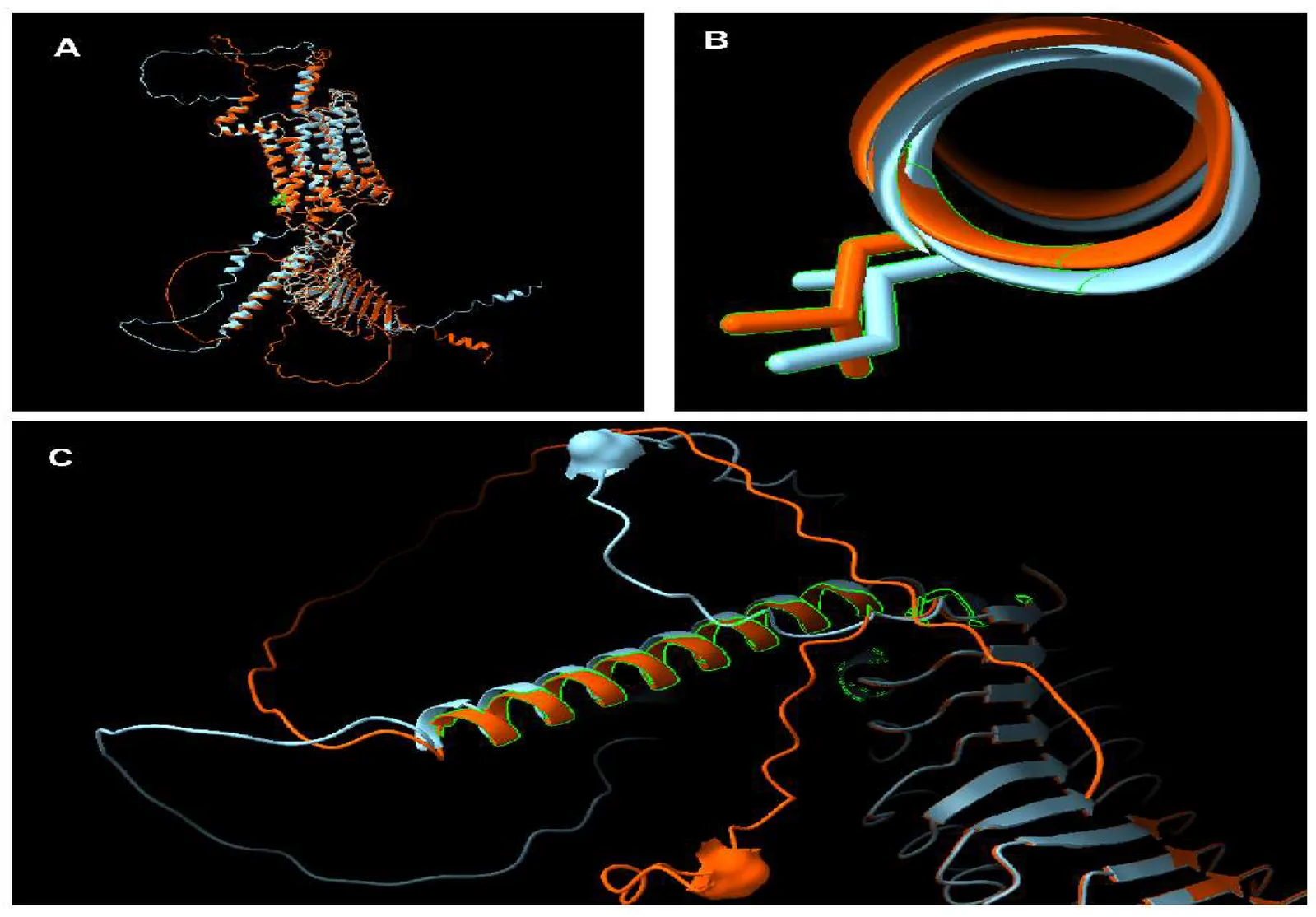
Comparative protein structure predictions for WT TSHR and the mutant T47D TSHR (**A**), the Ile419Leu mutation site (**B**) Ala380Pro mutation site (**C**) T47D structure is highlighted in orange and the WT structure in blue. The mutation position is highlighted in green.

**Figure 6:**
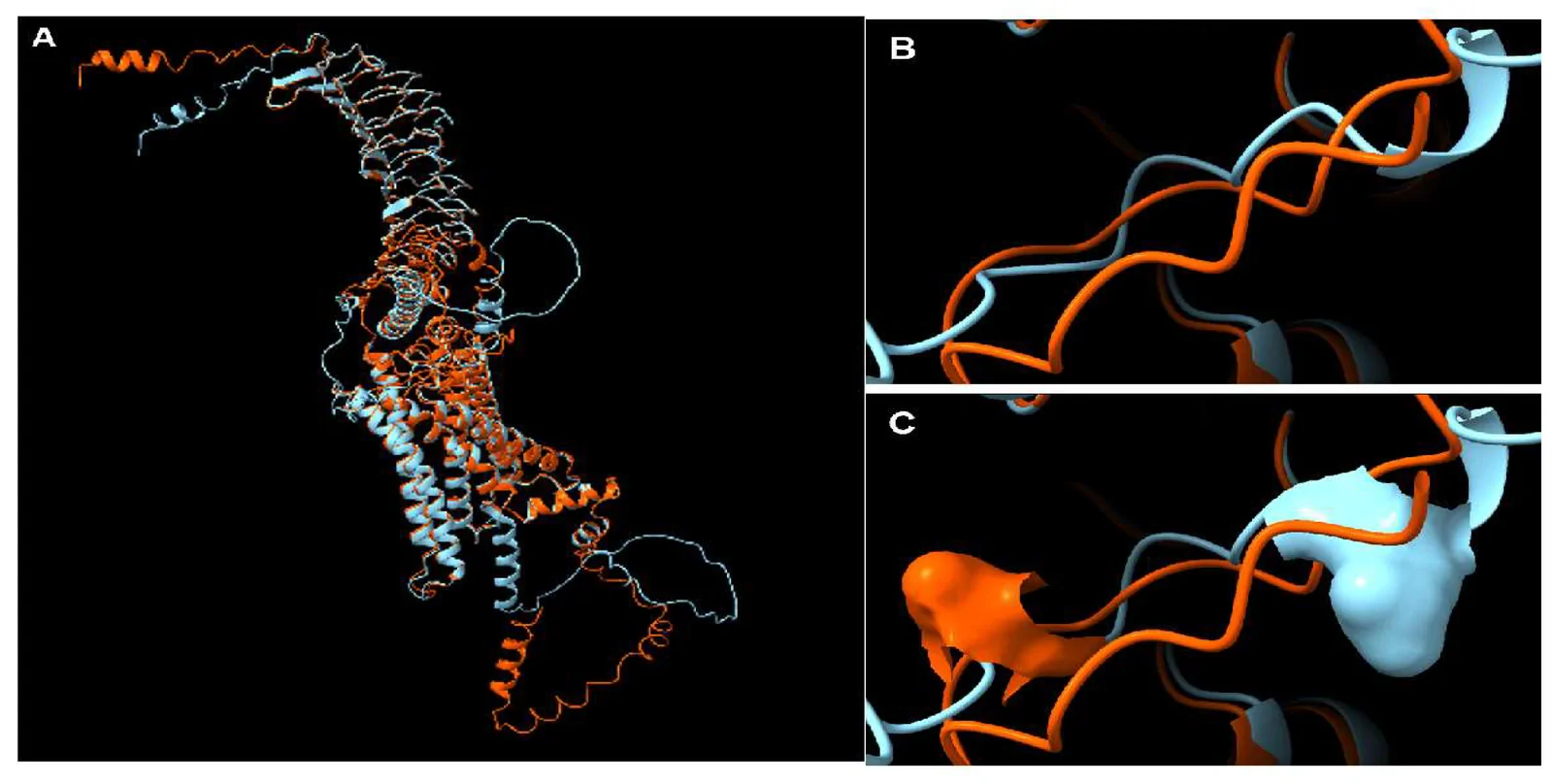
Comparative protein structure of TSHR predictions. WT TSHR in blue and the mutant PL45 and HCT116 TSHR in orange (**A**), the mutation and the WT site of Glu362Asp structure comparison (**B**) Pocket Expansion of the Glu362 site vs 362Asp site (**C**).

**Table 1:** Identified mutations in TSHR gene in cancer cell lines.

Cell Type	Position	Nucleotide Change (cDNA Position)	Amino Acid Change	Type of Mutation
PL45	Chr14, exon 10	c.1086 G > T	Glu 362 Asp (E362D)	Missense mutation/Polymorphism-heterozygous
PL45	Chr14, exon 10	c. 1257 T > A	Ile 419 Ile (I419I)	Silent mutation/Polymorphism-heterozygous
T47D	Chr14, exon 10	c. 1138 G > C	Ala 380 Pro (A380P)	Missense mutation
T47D	Chr14, exon 10	c. 1255 A > C c. 1257 T > A	Ile 419 Leu (I419L)	Missense mutation
T47D	Chr14, exon 10	c. 2181 C > G	Asp 727 Glu (D727E)	Missense mutation
HCT116	Chr14, exon 10	c.1086 G > T	Glu 362 Asp (E362D)	Missense mutation/Polymorphism-heterozygous
HCT116	Chr14, exon 10	c.1650 C > T	Phe 550 Phe (F550F)	Silent mutation/Polymorphism-heterozygous

**Table 2:** TSHR RT-PCR and TSHR cDNA sequencing primers.

Gene	Sequence	Orientation	Product Size (bp)
Primers for Real-Time PCR Reaction			
GAPDH	5′-CCTGCACCACCAACTGCTTA-3′ 5′-CCATCCACAGTCTTCTGAG–3′	F R	
TSHβ	5′-ATGCTCTGTCCCAGGATGTT-3′ 5′-TAGGGAGCAACATGGAGTGG-3′	F R	138
TSHR	5′-CTCCTCACCAGCCACTACAA-3 5′-CATCCCCATGCAGAAATCCG-3′	F R	81
Primers for TSHR Extragonadal Expression			
Band 2	5′-AAAATGAGGCCGGCGGACTTGC-3′ 5′-CCCTCCTAAAGTTCCTTGGC-3′	F R	416
Band 3	5′-AGACCCTGATGCCCTCAAAG-3′ 5′-CCTTCACCTCACACGGGCTG-3′	F R	476
Band 4	5′-AGGAACTGATAGCAAGAAACAC-3′ 5′-CATCCCCATGCAGAAATCCG-3′	F R	647
Band 5	5′-CTCCTCACCAGCCACTACAA-3′ 5′-CCATTGTGAGTAGTGTAGTGTTAAC-3′	F R	950
Band 7	5′-GTAAACGCCAGGCTCAGG-3′ 5′-CCATTGTGAGTAGTGTAGTGTTAAC-3′	F R	1057
Band 9	5′-AGACCCTGATGCCCTCAAAG-3′ 5′-CATCCCCATGCAGAAATCCG-3′	F R	225

## Data Availability

The data presented in this study are available on request from the corresponding author.

## Supplementary Materials

The following supporting information can be downloaded at: https://www.mdpi.com/article/10.3390/ijms27198487/s1.
