# Supplementary material for: Extrathyroidal Expression of the Thyrotropin Receptor in Epithelial Cancer Cells
Source: Int J Mol Sci. 2026 Sep 23;27(19):8487. doi: 10.3390/ijms27198487 (PMC13665271; doi:10.3390/ijms27198487)
Supplement: Supplementary file 1 [file ijms-27-08487-s001.zip › ijms-4551912-supplementary.pdf]

Supplement

Cell type	Nucleotide change (cDNA position)	Amino acid change	DNA sequence
PL45	c.1086 G>T	Glu 362 Asp (E362D)	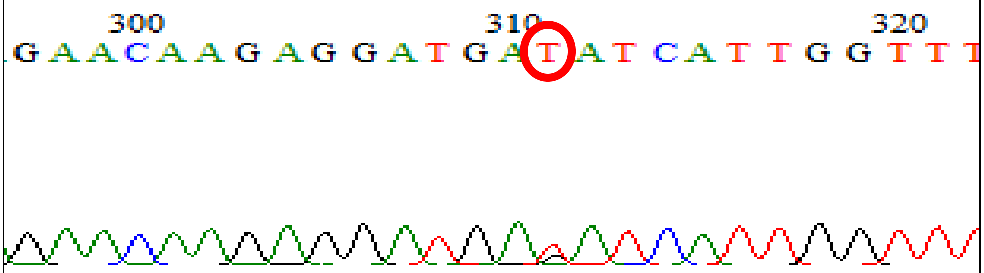
PL45	c. 1257 T>A	Ile 419 Ile (I419I)	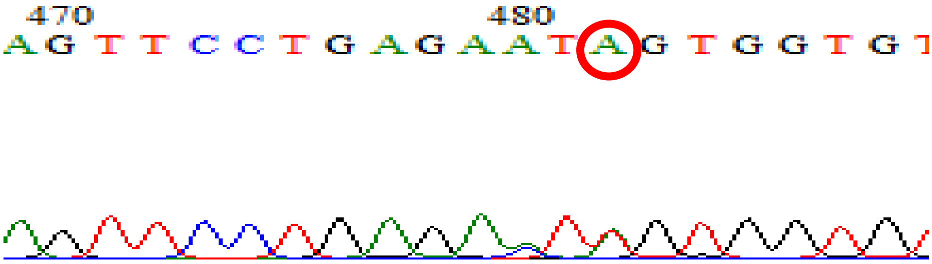
T47D	c. 1138 G>C	Ala 380 Pro (A380P)	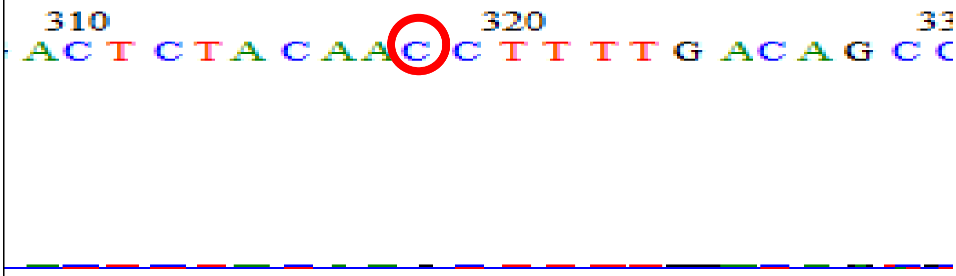
T47D	c. 1255 A>C<br>c. 1257 T>A	Ile 419 Leu (I419L)	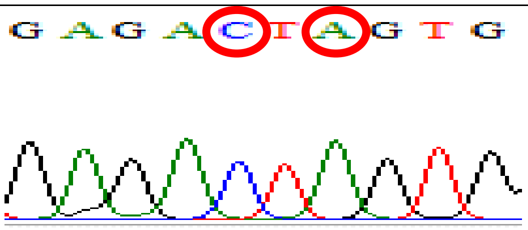

Supplement

T47D	c. 2181 C>G	Asp 727 Glu (D727E)	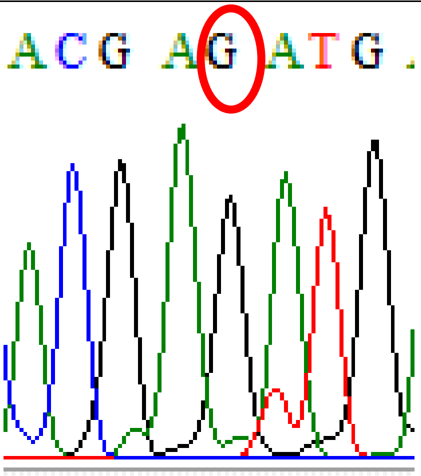
HCT116	c.1086 G>T	Glu 362 Asp (E362D)	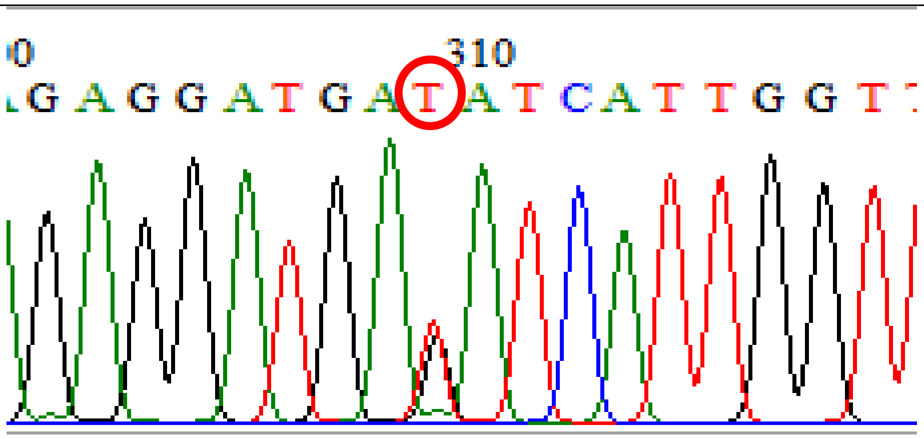
HCT116	c.1650 C>T	Phe 550 Phe (F550F)	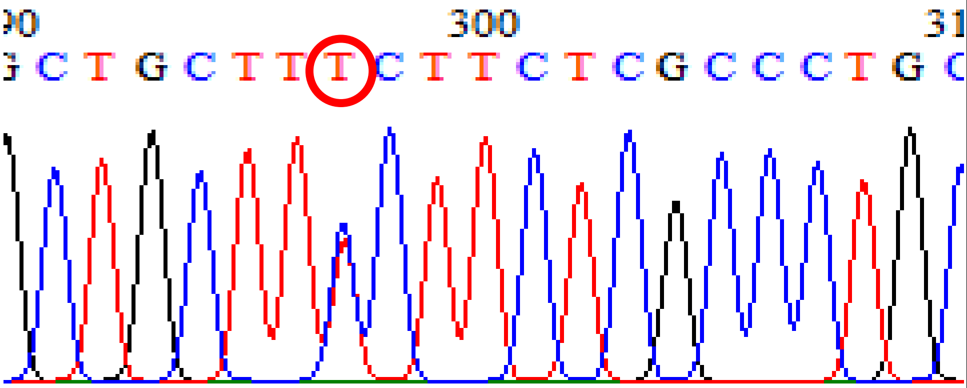

Figure S1. Identification of TSHR mutations by Sanger sequencing. cDNA was generated from total RNA isolated from PL45, T47D, and HCT116 cell lines by reverse transcription using oligo(dT) primers. The resulting cDNA was amplified by PCR using TSHR-specific primers, and the PCR products were subsequently subjected to Sanger sequencing by Macrogen (Amsterdam, The Netherlands). The obtained sequences were analyzed using Gene Runner and compared with the reference **TSHR** sequence using the NCBI BLAST database to identify sequence variants..
